# Joint Resource Optimization in Simultaneous Wireless Information and Power Transfer (SWIPT) Enabled Multi-Relay Internet of Things (IoT) System

**DOI:** 10.3390/s19112536

**Published:** 2019-06-03

**Authors:** Weidang Lu, Guangzhe Liu, Peiyuan Si, Guanghua Zhang, Bo Li, Hong Peng

**Affiliations:** 1 College of Information Engineering, Zhejiang University of Technology, Hangzhou 310014, China; luweid@zjut.edu.cn (W.L.); zjut_lgz@163.com (G.L.); a18158504979@163.com (P.S.); ph@zjut.edu.cn (H.P.); 2 School of Electrical Engineering and Information, Northeast Petroleum University, Daqing 163318, China; 3 School of Information and Electrical Engineering, Harbin Institute of Technology, Weihai 264209, China; libo1983@hit.edu.cn

**Keywords:** internet of things, SWIPT, orthogonal frequency division multiplexing (OFDM), multi-relay, decode-and-forward

## Abstract

The internet of things (IoT) is becoming more indispensable in modern society as the further development and maturity of information technology progresses. However the exponential growth of IoT devices leads to severe energy consumption. As a technology with broad application prospects, simultaneous wireless information and power transfer (SWIPT) enables IoT devices to harvest energy from receiving radio frequency (RF) signals while ensuring information transmission. In this paper, we investigate the transmission rate optimization problem for a dual-hop multi-relay IoT system, where a decode-and-forward (DF) relay supports the SWIPT technique. We jointly optimize the resource including power and subcarrier allocation, to maximize the system transmission rate. The time-sharing strategy and Lagrange dual method are used to solve this optimization problem. Simulation results reveal that the proposed algorithm has a larger transmission rate than other benchmark algorithms when ensuring each relay has no additional energy supply. Specifically, the proposed algorithm improves the information transmission rate by 2.8%, 3.4% and 43% compared with other algorithms in the case of five relays when the source’s power is equal to 0.5 W, respectively.

## 1. Introduction

In recent years, breakthrough progress has been made in the internet of things (IoT) technique which has been widely applied in industrial automation, intelligent medicine, smart grids, etc. [1,2,3]. According to estimates, the communication system will support more than 50 billion IoT devices by 2015 [4]. In the meantime, these put forward larger requirements on energy supplies [5]. Therefore it is extremely urgent to design a green sustainable scheme which provides a larger transmission rate without requiring frequent battery replacements [6].

Compared to the traditional battery energy supplies, energy harvesting (EH) technology can extract renewable energy from the surrounding environment, which is not always stable and controllable. Magnetic induction and radio frequency (RF) are two common ways to realize wireless power transfer, which can supply stable energy. However, the power transfer range will be very limited through magnetic induction [7,8]. With the continuous breakthrough of ultra-low-power semiconductors, RF plays a more important role in long-distance communication [9,10], which has the ability to carry both information and energy. Therefore, as a technology with broad application prospects, simultaneous wireless information and power transfer (SWIPT) has roused great concern from academia, and has been widely investigated [11,12,13,14,15].

In the SWIPT enabled IoT system, extending the radio coverage and increasing spectrum utilization are two key points of the design. Cooperative relaying technology has been widely applied by virtue of its ability to extend the coverage of radio waves, reduce fading, and increase system capacity [16,17,18,19]. However, relays require to have battery storage capability or external charging to keep running, which is not conducive to flexible placement of relays. The SWIPT technology can solve the energy limitation problem of relays. Therefore, the introduction of cooperative relaying technology into a SWIPT enabled IoT system is an effective way [20]. The author in [21] investigates the spectrum efficiency optimization problem in a cognitive radio system with SWIPT, where IoT devices serve as relays operating decode-and-forward (DF) to assist the system’s information interaction. A joint optimization design for SWIPT-enabled IoT network with a DF relay is studied in [22], where the reliability is improved by combining the advantages of power-splitter (PS) and time-splitter (TS). To achieve the balance between energy efficiency and spectral efficiency, a locally optimal power allocation scheme is designed in [23] for SWIPT amplify-and-forward (AF) relaying networks, which reduces computational complexity and maintains high performance.

Efficient utilization of spectrum is another emphasis for IoT design. Due to the high spectrum efficiency of orthogonal frequency division multiplexing (OFDM) and its resistance to multipath fading [24,25], the design combining OFDM and SWIPT for IoT system becomes a promising way to enable higher data demands and lower energy requirements. In [26], a multiuser scenario was considered in an OFDM-based SWIPT system, where the optimal rate is obtained by iteratively adjusting resource allocation under the condition of meeting the energy harvesting requirement. In [27], two suboptimal algorithms were provided for a downlink SWIPT based orthogonal frequency division multiple access (OFDMA) network with the goal of maximizing the energy harvested, which achieves the balance between reducing complexity and maintaining system performance. In [28], a new receiver architecture was designed which employs SWIPT technology and reduces the energy required for decoding. This scheme expands the rate-energy boundary greatly.

Therefore, combining relaying cooperation technology and OFDM technology with SWIPT in IoT systems is a promising scheme which can enable higher data demands effectively while meeting the design requirements of spectrum and radio coverage. Some research has been done in this field and researchers abroad have made great progress. In view of the availability of the link between source and destination in an OFDM SWIPT system, Ref. [29] proposes two protocols to achieve the maximum system transmission rate, where the relay adopting PS method harvests energy and transmits information simultaneously. Ref. [30] considers an AF-OFDM relaying system with SWIPT, which jointly optimizes multiple resources to achieve higher system performance while reducing computational effort.

The aforementioned relaying SWIPT IoT systems are based on single relay. To the best of our knowledge, SWIPT has not been applied in the multi-relay OFDM based IoT system, which motivates our study. Thus, in this paper we propose a joint resource optimization scheme in a SWIPT enabled multi-relay OFDM based IoT system to obtain the maximum transmission rate, in which the transmission between the source and the destination is carried out through the assistance of multiple SWIPT enabled DF relays.

The main contributions of this paper can be summarized as follows:

- We consider a multi-relay OFDM based IoT system, in which the SWIPT-enabled relay can decode information and harvest energy simultaneously. Therefore, the splitter does not have to be installed in the relay, which is necessary in the common algorithm using PS or TS protocol. It can simplify the deployment and administration of the IoT system.
- We formulate a scheme by optimizing power and subcarrier allocation with the aim at maximizing the transmission rate, and employ the time-sharing strategy and Lagrange dual method to solve this optimization problem.
- Simulation results illustrate the effectiveness of our algorithm. By observing the transmission rate, it illustrates that the proposed algorithm has better performance than other algorithms.

## 2. Review Conclusion

In SWIPT systems, TS and PS are the two most commonly used methods in receivers because of their ease of implementation. In [20,27,28], the PS based receiver divides the received signal stream into two parts by the power splitter, thereby realizing the synchronization of information transmission and energy harvesting. In [21,23,31], the TS based receiver uses the time splitter to set the energy harvesting and information transmission operations to be performed at different timeslots. In this paper, we utilize the characteristics of OFDM through different subcarriers to implement SWIPT without splitters, thus, there is no need to optimize PS/TS ratios, which is necessary in the aforementioned literature for better system performance.

As mentioned in the introduction section, researchers have proposed many advanced strategies to improve energy efficiency, such as the three-step design in [22], the transmission mode adaptation in [29], and the multi-dimensional resource optimization framework in [30]. However, these do not have applicability in the case of multiple relays having the ability to improve the system capacity and diversity gain [32]. Our proposed algorithm can solve the energy limitation problem of multi-relay IoT system effectively.

The rest of the paper is organized as follows. In Section 3, we introduce the system model and present the problem formulation. The proposed joint resource allocation solution is illustrated in Section 4. Section 5 provides simulation results and discussions. Finally, conclusions are drawn in Section 6.

## 3. System Model and Problem Formulation

**Figure 1:**
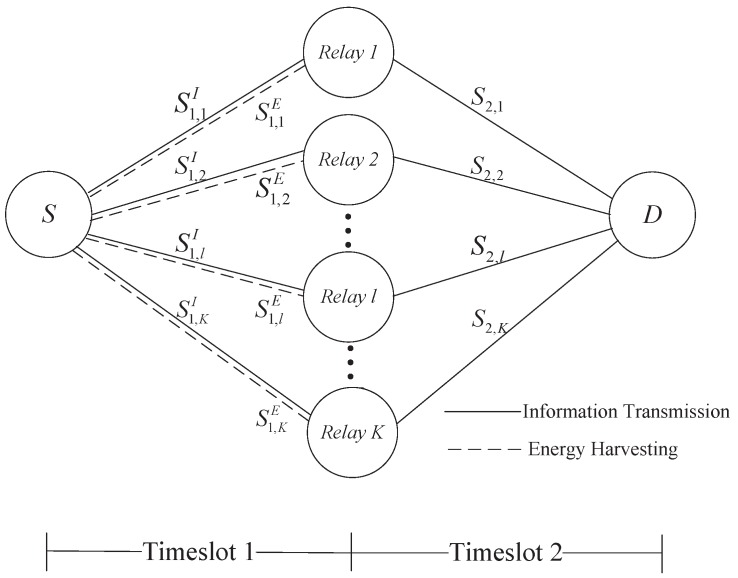
System model. Source *S*, destination *D*, and *K* relays.

As shown in Figure 1, we consider a dual-hop SWIPT enabled multi-relay OFDM based IoT system, where relays operate in time-division half-duplex mode based DF protocol. This system consists of one source *S*, one destination *D*, and *K* relays. Let $K=\left\{1,2,\ldots ,K\right\}$ denote the set of *K* number of relays. The set of *N* subcarriers is denoted by $N=\left\{1,2,\ldots ,N\right\}$. Due to the limited coverage and shadowing attenuation, the direct link between *S* and *D* is infeasible. In addition, perfect channel state information (CSI) is available for acquisition.

The transmission between *S* and *D* are performed through the following two timeslots. In the first timeslot, *S* broadcasts the signal over all the subcarriers. $n\in S_{1,k}^{I}$ denotes the subcarriers allocated by relay *k*$\left(k\in K\right)$ for decoding the received information, and $n\in S_{1,k}^{I}$ denotes the remaining subcarriers which are utilized to harvest energy. In the second timeslot, relay *k* forwards the re-encoded information over the subcarriers $n\in S_{2,k}$ to *D*. In order to prevent mutual interference, a subcarrier utilized for information transmission will be only assigned to one relay in each timeslot.

Let the channel gain and the allocated power on subcarrier *n* associated with relay *k* in hop *t* be denoted as $h_{n,k,t}$ and $p_{n,k,t}$ respectively, for n∈N, k∈K and $t=\left\{1,2\right\}$. Thus, the transmission rate $r_{n,k,t}$ can be expressed as

(1) $$r_{n,k,t}=\ln\left(1+\frac{h_{n,k,t}p_{n,k,t}}{\sigma^{2}}\right)$$

where $\sigma^{2}$ represents the variance of additive white Gaussian noise and $p_{n,k,1}$ is equally allocated.

The harvested energy at relay *k* is given by

(2) $$Q_{k}=\sum_{n\in S_{1,k}^{E}}\xi \left(h_{n,k,1}p_{n,k,1}+\sigma^{2}\right)$$

where ξ is the energy conversion efficiency of relays.

The transmission rate from source to destination via relay *k* is the minimum of the rate achieved over two hops, which can be written as

(3) $$r_{k}=\frac{1}{2}\min\left(\sum_{n\in S_{1,k}^{I}}r_{n,k,1},\sum_{n\in S_{2,k}}r_{n,k,2}\right).$$

Our goal is to maxmize the system transmission rate through optimizing the joint resource allocation, including power allocation $p=\left\{p_{n,k,2}\right\}$ and subcarrier allocation $\Omega =\left\{S_{1,k}^{I},S_{1,k}^{E},S_{2,k}\right\}$. Thus, the optimization problem can be mathematically formulated as

$$\begin{array}{cc} (P1):\; & \;\max_{\left\{p,\Omega \right\}}\;\;\sum_{k=1}^{K}r_{k} \end{array}$$

(4) $$\begin{array}{cc} s.t. & C1:Q_{k}\geq \sum_{n\subseteq S_{2,k}}p_{n,k,2},\forall k \end{array}$$

(5) $$\begin{array}{c} C2:S_{1,k}^{I}\cap S_{1,k^{\prime }}^{I}=⌀,\forall k\neq k^{\prime } \end{array}$$

(6) $$\begin{array}{c} C3:S_{2,k}\cap S_{2,k}=⌀,\forall k\neq k^{\prime } \end{array}$$

(7) $$\begin{array}{c} C4:S_{1,k}^{I}\cap S_{1,k}^{P}=⌀,\forall k \end{array}$$

(8) $$\begin{array}{c} C5:S_{1,k}^{I}+S_{1,k}^{P}=N,\forall k \end{array}$$

(9) $$\begin{array}{c} C6:p_{n,k,2}\geq 0,\forall n,k \end{array}$$

where C1 represents the energy used by each relay to forward *S* signal in the second timeslot should be not larger than the energy it harvests in the first timeslot, and C2∼C5 indicate the constraints of the subcarrier set.

## 4. Optimal Resource Allocation

In order to reduce the computational complexity of P1 which is a mixed integer programming problem, we apply the time-sharing strategy which introduces the factors $\left\{\tau_{n,k,t}\right\}$, for n∈N, k∈K, t=1,2. We assume that $\tau_{n,k,i}$ indicates a part of time that subcarrier *n* is allocated to relay *k* over hop *t*. The variables $\left\{\tau_{n,k,t}\right\}$ satisfy $\sum_{k}^{K}\rho_{n,k,t}=1$, ∀n,t. It is proved in [33,34] that the optimal results obtained by the time-sharing strategy are almost identical to those achieved by integer channel allocations. Hence, the optimization problem can be rewritten by

$$\begin{array}{cc} (P2)\text{:}\; & \;\max_{\left\{\tau ,p\right\}}\;\;\sum_{k=1}^{K}r_{k} \end{array}$$

(10) $$\begin{array}{cc} s.t. & C7:\sum_{n=1}^{N}\tau_{n,k,t}\ln\left(1+\frac{h_{n,k,t}p_{n,k,t}}{\sigma^{2}}\right)\geq r_{k},\forall k,t \end{array}$$

(11) $$\begin{array}{c} C8:\sum_{n=1}^{N}\left(1-\tau_{n,k,1}\right)\xi \left(h_{n,k,1}p_{n,k,1}+\sigma^{2}\right)\geq \sum_{n=1}^{N}\tau_{n,k,2}p_{n,k,2},\forall k \end{array}$$

(12) $$\begin{array}{c} C9:\sum_{k=1}^{K}\tau_{n,k,t}=1,\forall n,t \end{array}$$

(13) $$\begin{array}{c} C2,C3,C4,C5,C6. \end{array}$$

The total variables to be optimized in P2 include subcarrier arrangement $\tau =\left\{\tau_{n,k,t}\right\}$, the power allocation p. Since the time-sharing condition is satisfied [35], the optimization problem is convex, thus, the Lagrange dual method can be utilized to obtain an asymptotically optimal solution. In the following sub-sections, we illustrate the detailed derivation of the optimal resource allocation by applying the dual method.

### 4.1. Optimizing the Dual Function

We use $s_{n,k,t}=\tau_{n,k,t}\cdot p_{n,k,t}$ to denote the actual consumption on the subcarrier corresponding to $\tau_{n,k,t}$. Define D as the set of all possible variables $\left\{s,\tau ,r\right\}$ satisfying the corresponding constraints of P2, $\tau_{n,k,t}\geq 0$, $s_{n,k,t}\geq 0$, and $r_{k}\geq 0$, where $r=\left\{r_{n,k,t}\right\}$. Then the Lagrange dual function of P2 is expressed as

(14) $$g\left(\alpha ,\beta \right)≜\max_{\left\{s,r,\tau \right\}\in D}\;\;L\left(s,r,\tau ,\alpha ,\beta \right)$$

where

(15) $$\begin{array}{cc} L\left(s,r,\tau ,\alpha ,\beta \right) & =\sum_{k=1}^{K}r_{k}+\sum_{t=1}^{2}\sum_{k=1}^{K}\alpha_{k,t}\left[\sum_{n=1}^{N}\tau_{n,k,t}\ln\left(1+\frac{h_{n,k,1}s_{n,k,t}}{\sigma^{2}\tau_{n,k,t}}\right)-r_{k}\right]\\ & +\sum_{k=1}^{K}\beta_{k}\left(\sum_{n=1}^{N}\left(1-\tau_{n,k,1}\right)\xi \left(h_{n,k,1}\frac{s_{n,k,1}}{\tau_{n,k,1}}+\sigma^{2}\right)-\sum_{n=1}^{N}s_{n,k,2}\right) \end{array}$$

where $\alpha =\left\{\alpha_{k,1},\alpha_{k,2}\right\}\geq 0$ and $\beta =\left\{\beta_{k}\right\}\geq 0$ are the non-negative Lagrange multipliers for the constraints (10), (11). The dual optimization problem is hence given by

(16) $$\begin{array}{c} \underset{\left\{\alpha ,\beta \right\}}{min}\;g\left(\alpha ,\beta \right)\\ s.t.\;\alpha \geq 0,\beta \geq 0. \end{array}$$

It can be proved by the definition of [36] this dual function is convex, thus, we can apply the subgradient method to obtain the optimal dual variables $\left\{\alpha^{*},\beta^{*}\right\}$ to minimize $g\left(\alpha ,\beta \right)$ with guaranteed convergence. Then a subgradient of $g\left(\alpha ,\beta \right)$ can be derived as below

(17) $$\begin{array}{c} \nabla \alpha_{k,t}=\frac{1}{2}\sum_{n=1}^{N}\tau_{n,k,t}\ln\left(1+\frac{h_{n,k,t}s_{n,k,t}}{\sigma^{2}\tau_{n,k,t}}\right)-r_{k},\forall k,t \end{array}$$

(18) $$\begin{array}{c} \nabla \beta_{k}=\sum_{n=1}^{N}\left(1-\tau_{n,k,1}\right)\xi \left(h_{n,k,1}\frac{s_{n,k,1}}{\tau_{n,k,1}}+\sigma^{2}\right)-\sum_{n=1}^{N}s_{n,k,2},\forall k. \end{array}$$

Therefore, the dual variables are updated as $\Gamma^{\left(m+1\right)}=\Gamma^{\left(m\right)}+\eta^{\left(m\right)}\Delta \Gamma$, where $\Delta \Gamma =\left(\Delta \beta_{1},\ldots ,\Delta \beta_{k},\Delta \alpha_{1,1},\ldots ,\Delta \alpha_{k,1},\Delta \alpha_{1,2},\ldots ,\Delta \alpha_{k,2}\right)$ and $\eta^{\left(m\right)}$ denotes the step size. Follow the diminishing step size policy in [37] to ensure that the optimal variables $\Gamma^{*}$ can be converged. The complexity of this method is $O\left({\left(3K\right)}^{\nu }\right))$, where ν is a nonnegative integer.

### 4.2. Optimizing Primal Variables at a Given Dual Point

After obtaining $\Gamma^{*}$, we need to determine the optimal $\left\{s^{*},\tau^{*}\right\}$ in this subsection. Observing (15), we can decompose the dual function as follows

(19) $$g\left(\alpha ,\beta \right)=g_{0}\left(\alpha \right)+\sum_{n=1}^{N}g_{n,1}\left(\alpha ,\beta \right)+\sum_{n=1}^{N}g_{n,2}\left(\alpha ,\beta \right)+\sum_{k=1}^{K}\beta_{k}\left(\sum_{n=1}^{N}\xi \left(h_{n,k,1}p_{n,k,1}+\sigma^{2}\right)\right)$$

where

(20) $$\begin{array}{cc} g_{0}\left(\alpha \right) & =\max_{r\in D}\;\;L_{0}\left(r,\alpha \right)=\max_{r\in D}\;\;\sum_{k=1}^{K}\left(1-\alpha_{k,1}-\alpha_{k,2}\right)\cdot r_{k} \end{array}$$

(21) $$\begin{array}{cc} g_{n,1}\left(\alpha ,\beta \right) & =\max_{\left\{s,\tau \right\}\in D}\;\;L_{n,1}\left(s,\tau ,\alpha ,\beta \right)\\ & =\max_{\left\{s,\tau \right\}\in D}\;\sum_{k=1}^{K}\left[\alpha_{k,1}\tau_{n,k,1}\ln\left(1+\frac{h_{n,k,1}s_{n,k,1}}{\sigma^{2}\tau_{n,k,1}}\right)-\beta_{k}\tau_{n,k,1}\xi \left(h_{n,k,1}\frac{s_{n,k,1}}{\tau_{n,k,1}}+\sigma^{2}\right)\right]\;,\forall n \end{array}$$

(22) $$\begin{array}{cc} g_{n,2}\left(\alpha ,\beta \right) & =\max_{\left\{s,\tau \right\}\in D}\;\;L_{n,2}\left(s,\tau ,\alpha ,\beta \right)\\ & =\max_{\left\{s,\tau \right\}\in D}\;\sum_{k=1}^{K}\left[\alpha_{k,2}\tau_{n,k,2}\ln\left(1+\frac{h_{n,k,2}s_{n,k,2}}{\sigma^{{}^{2}}\tau_{n,k,2}}\right)-\beta_{k}s_{n,k,2}\right]\;,\forall n. \end{array}$$

It can be observed that $g_{0}\left(\alpha \right)$ is linearly correlated with $r_{k}$. In order to maximize $L_{0}\left(r,\alpha \right)$, the optimal $r_{k}^{*}$ must satisfy

(23) $$r_{k}^{*}=\left\{\begin{array}{c} 0,\;if\;\alpha_{k,1}+\alpha_{k,2}>1\\ any,if\;\alpha_{k,1}+\alpha_{k,2}=1,\forall k\in K\\ \infty ,\;\;if\;\alpha_{k,1}+\alpha_{k,2}<1 \end{array}\right.$$

If $\alpha_{k,1}+\alpha_{k,2}<1$, $g\left(\alpha ,\beta \right)$ tends to infinity which means it cannot be minimized. Therefore, the optimal dual variables must satisfy the constraint of $\alpha_{k,1}+\alpha_{k,2}\geq 1$, while $g_{0}\left(\alpha \right)$ identically equal to 0. Through the following two steps, we can obtain the optimal $\left\{p,\tau \right\}$ with the optimal variables $\Gamma^{*}$.

(1)Optimal Power Allocation for Given Subcarrier Allocation: As $L_{n,2}\left(s,\tau ,\alpha ,\beta \right)$ is a concave function of $s_{n,k,2}$, we can get the optimal power allocation by applying the Karush-Kuhn-Tucker (KKT) condition [36]. More specifically, calculating the derivative of function (22) with respect to $s_{n,k,2}$ and making it equal to 0. The optimal power $p_{n,k,2}^{*}$ are given by

(24) $$p_{n,k,2}^{*}=\frac{s_{n,k,2}^{*}}{\tau_{n,k,2}}={\left(\frac{\alpha_{n,2}}{\beta_{k}}-\frac{\sigma_{n,k,2}^{2}}{h_{n,k,2}}\right)}^{+}$$

where ${\left(x\right)}^{+}=\max\left\{0,x\right\}$. The complexity of calculating the $p_{n,k,2}^{*}$ is O(1).

(2)Optimal Subcarrier Allocation: Substituting the obtained power allocation into Equations (21) and (22) respectively, we have

(25) $$\begin{array}{cc} \;L_{n,1}\left(\tau ,\alpha ,\beta \right) & =\sum_{k=1}^{K}\left[\alpha_{k,1}\tau_{n,k,1}\ln\left(1+\frac{h_{n,k,1}P_{s}}{\sigma^{2}N}\right)-\beta_{k}\tau_{n,k,1}\xi \left(h_{n,k,1}\frac{P_{s}}{N}+\sigma^{2}\right)\right]\;,\forall n \end{array}$$

(26) $$\begin{array}{cc} L_{n,2}\left(\tau ,\alpha ,\beta \right) & =\sum_{k=1}^{K}\left[\alpha_{k,2}\tau_{n,k,2}\ln\left(\frac{h_{n,k,2}\alpha_{n,2}}{\sigma^{2}\beta_{k}}\right)-\beta_{k}\tau_{n,k,2}\left(\frac{\alpha_{n,2}}{\beta_{k}}-\frac{\sigma^{2}}{h_{n,k,2}}\right)\right],\;\forall n \end{array}$$

where $P_{s}$ is the source’s transmitted power.

By extracting the common factors $\tau_{n,k,1}$ and $\tau_{n,k,2}$ respectively, Equations (25) and (26) can be expressed as

(27) $$L_{n,t}\left(\tau ,\alpha ,\beta \right)=\sum_{k=1}^{K}\rho_{n,k,t}H_{n,k,t},\;t=1,2$$

where

(28) $$\begin{array}{cc} H_{n,k,1} & =\alpha_{k,1}\ln\left(1+\frac{h_{n,k,1}P_{s}}{\sigma^{2}N}\right)-\beta_{k}\xi \left(h_{n,k,1}\frac{P_{s}}{N}+\sigma^{2}\right) \end{array}$$

(29) $$\begin{array}{cc} H_{n,k,2} & =\alpha_{k,2}\ln\left(\frac{h_{n,k,2}\mu_{n,2}}{\sigma^{2}\beta_{k}}\right)-\beta_{k}\left(\frac{\mu_{n,2}}{\beta_{k}}-\frac{\sigma^{2}}{h_{n,k,2}}\right). \end{array}$$

Based on the result of the above analysis, we can finally get

(30) $$g\left(\alpha ,\beta \right)=\sum_{k=1}^{K}\beta_{k}\left(\sum_{n=1}^{N}\xi \left(h_{n,k,1}\frac{P_{S}}{N}+\sigma^{2}\right)-P_{r}\right)+\sum_{i=1}^{2}\sum_{n=1}^{N}\left(\max\sum_{k=1}^{K}\tau_{n,k,i}H_{n,k,i}\right).$$

It can be concluded that in order to maximize $g\left(\alpha ,\beta \right)$, the optimal time-sharing parameter $\left\{\tau_{n,k,t}^{*}\right\}$ should have the largest $\sum_{k=1}^{K}\tau_{n,k,t}H_{n,k,t}$ for subcarrier *n* in hop *t*. As stated in Section 3, the optimal solution obtained using the time-sharing strategy approximates the optimal goal obtained utilizing integer channel allocation optimization. According to the constraints of (12) and (13), if the subcarrier *n* over hop *t* is allocated to the relay *k*, then $\tau_{n,k,t}=1$, otherwise $\tau_{n,k^{\prime },t}=0$, for $k^{\prime }\neq k$. It can also be observed that subcarriers for information transmission in the same timeslot are independent of each other. Therefore, in ensuring the validity of Lagrange dual method, the optimal subcarrier allocation is given by

(31) $$\tau_{n,k,t}^{*}=\left\{\begin{array}{c} 1,\;\mathrm{if}\;k=\arg\max_{k}\left\{H_{n,k,t}\right\}\\ 0,\;\mathrm{otherwise}. \end{array}\right.$$

**Figure 2:**
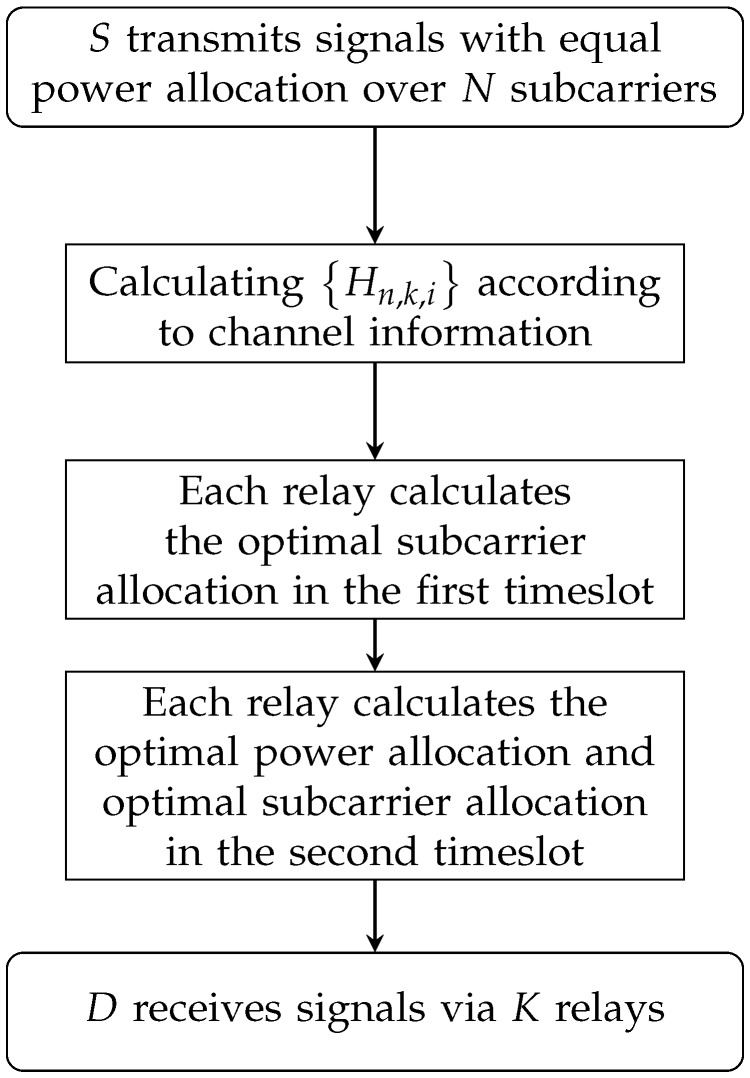
Flowchart of the proposed algorithm.

The complexity of calculating the $\tau_{n,k,t}^{*}$ is O(NK). Therefore, the proposed algorithm complexity solving P2 is $O\left({\left(3K\right)}^{\nu }\cdot 1\cdot NK\right)=O\left(NK{\left(3K\right)}^{\nu }\right)$. The flowchart representing the logic of our algorithm and its pseudocode are shown in Figure 2 and Algorithm 1.

**Algorithm 1** Proposed Algorithm for **P2**
1:**Initialize**$\left\{\beta_{k},\alpha_{k.t}\right\}$, and given $P_{S}$.2:
**Repeat**
3: Compute the optimal power allocations $\left\{p_{n,k,2}^{*}\right\}$ in (24) and $\left\{H_{n,k,i}\right\}$ in (28), (29).4: Obtain the optimal subcarrier allocations $\left\{S{{}_{1,k}^{I}}^{*},S{{}_{1,k}^{E}}^{*},{S_{2,k}}^{*}\right\}$ according to (31).5: Update $\left\{\beta_{k},\alpha_{k.t}\right\}$ by the subgradient method defined in (17), (18).6: **Until**$\left\{\beta_{k},\alpha_{k.t}\right\}$ converge.


## 5. Simulation Results and Analysis

In this section, simulation results are illustrated to evaluate the performance of the proposed algorithm. We consider the Rayleigh fading channel with the central frequency given at 1.9 GHz. We set the distance from the source to each relay and the distance from each relay to the destination both as 2 m. The number of subcarriers is 32, and the noise variance is fixed to −80 dbm.

**Figure 3:**
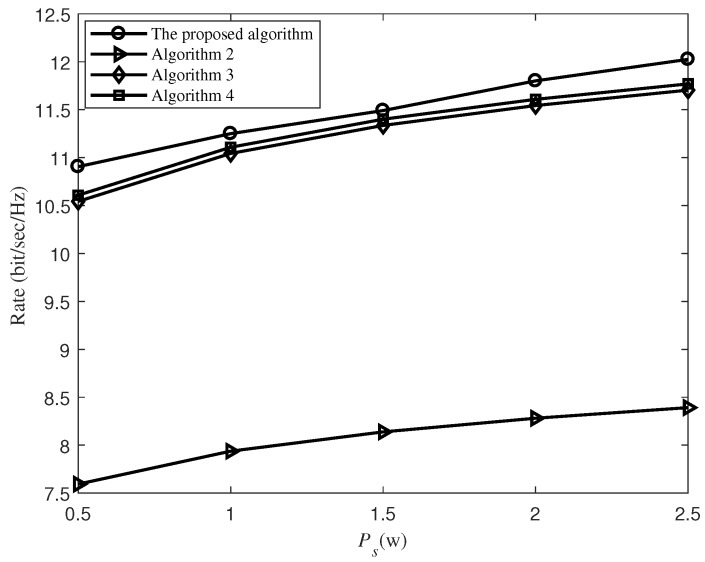
The transmission rate versus the source transmit power $P_{s}$.

In order to demonstrate the superiority of our proposed algorithm, Figure 3 exhibits the performance comparison of the proposed algorithm with the following three algorithms as shown in Algorithms 2–4, where there are five relays and the energy conversion efficiency at each relay is set as ξ=1.

Algorithm 2: The power allocation for relay to forward the *S* signal in the second timeslot is equally allocated. The subcarrier allocation is performed according to $k_{n,t}^{*}=\arg\max_{k}\left\{SNR_{n,k,t}\right\}$, where $SNR_{n,k,t}=\frac{h_{n,k,t}p_{n,k,t}}{\sigma^{2}}$, which means in the hop *t*, the subcarrier *n* will be allocated to relay *k* which has the maximum value of $SNR_{n,k,t}$.


**Algorithm 2**

1:Given $P_{S}$.2:Compute ${SNR}_{n,k,t}$.3:Obtain the optimal subcarrier allocations $\left\{S{{}_{1,k}^{I}}^{*},S{{}_{1,k}^{E}}^{*},{S_{2,k}}^{*}\right\}$ according to $k_{n,t}^{*}=\arg\max_{k}\left\{SNR_{n,k,t}\right\}$.4:Compute the power allocation $p_{n,k,2}=\sum_{n\in S{{}_{1,k}^{E}}^{*}}\xi \left(h_{n,k,1}p_{n,k,1}+\sigma^{2}\right)/\left|G_{S{{}_{2,k}}^{*}}\right|$.


Algorithm 3: The power allocation for relay to forward the *S* signal in the second timeslot is equally allocated. In the first timeslot, subcarriers are equally allocated to *K* relays. And in the second timeslot, relays utilized the same subcarriers to forward the *S* signal.


**Algorithm 3**

1:**Initialize**$S_{1,k}^{I}=⌀$ and given $P_{S}$.2:**For**i=1 to $\frac{N}{K}$3:$S_{1,k}^{I}=\left\{S_{1,k}^{I},n+\frac{N\left(k-1\right)}{K}\right\}$.4:
**end**
5:$S_{2,k}=S_{1,k}^{I}$ and $S_{1,k}^{E}=N-S_{1,k}^{I}$.6:Compute the power allocation $p_{n,k,2}=\sum_{n\in S_{1,k}^{E}}\xi \left(h_{n,k,1}p_{n,k,1}+\sigma^{2}\right)/\left|G_{S_{2,k}}\right|$.


Algorithm 4: The power allocation for a relay to forward the *S* signal in the second timeslot is equally allocated. The number of subcarriers for each relay used to decode information in the first timeslot is consistent with the number of subcarriers utilized for forwarding re-encoded information in the second timeslot. The Hungary method is used to perform the subcarrier allocation according to $u_{n,n^{\prime },k}=\frac{SNR_{n,k,1}+SNR_{n^{\prime },k,2}}{SNR_{n,k,1}\cdot SNR_{n^{\prime },k,2}}$, where $SNR_{n,k,1}=\frac{h_{n,k,1}p_{n,k,1}}{\sigma^{2}}$ and $SNR_{n^{\prime },k,2}=\frac{h_{n^{\prime },k,2}p_{n^{\prime },k,2}}{\sigma^{2}}$.


**Algorithm 4**

1:Given $P_{S}$.2:Compute $u_{n,n^{\prime },k}$.3:Obtain the optimal subcarrier allocation $\left\{S{{}_{1,k}^{I}}^{*},S{{}_{1,k}^{E}}^{*},{S_{2,k}}^{*}\right\}$ using the Hungary method.4:Compute the power allocation $p_{n,k,2}=\sum_{n\in S{{}_{1,k}^{E}}^{*}}\xi \left(h_{n,k,1}p_{n,k,1}+\sigma^{2}\right)/\left|G_{S{{}_{2,k}}^{*}}\right|$.


As shown in Figure 3, the proposed algorithm has performance advantages in comparision with the other three algorithms. It can be found that the performance of Algorithms 3 and 4 is close to our proposed algorithm, which is because that the transmission rate assisted by the relays is determined by the smaller of rates realized over the two hops, and they guarantee that the number of subcarriers assigned by each relay for information transmission is the same as the number of subcarriers used for forwarding the signal, which reduces the negative impact of unreasonable power allocation. Algorithm 2 achieves the worst performance because it depends entirely on the unstable signal to noise ratio (SNR), which is subject to random channel interference. In particular, when $P_{s}=0.5$ W, our proposed algorithm achieves improvement in the transmission rate of about 2.8%, 3.4% and 43% over Algorithms 2 to 4. It can also be seen from Figure 3 that the transmission rate of all algorithms becomes larger when $P_{s}$ increases. It is because more energy can be harvested from subcarriers when $P_{s}$ increases, which will achieve a larger information transmission rate in the second timeslot.

**Figure 4:**
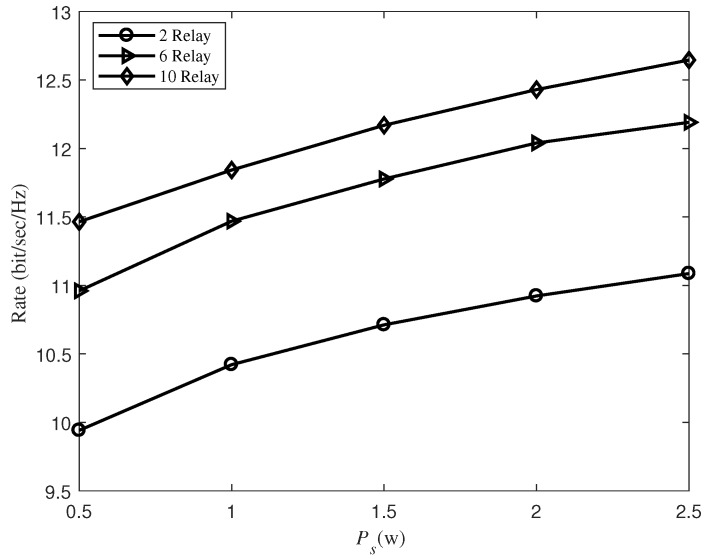
The transmission rate versus the source transmit power $P_{s}$ with different number of relays.

Figure 4 depicts the transmission rate versus $P_{s}$ with different number of relays. In Figure 4, we find that when the number of relays increase, the achieved rates also increase. For example, when $P_{S}=2.5$ W, the transmission rate achieved by six relays and 10 relays is increased by about 1.1 bit/s/Hz and 1.6 bit/s/Hz, respectively, compared to the case of two relays. The reason is that with the increase of relays, it can have more and better channels, then each relay will assign more subcarriers for energy harvesting. Moreover, it is shown that the transmission rate decreases as the transmission power decreases with the same number of relays. In particular, at six relays, the transmission rate dropped by 10% as the source’s power dropped from 2.5 W to 0.5 W.

**Figure 5:**
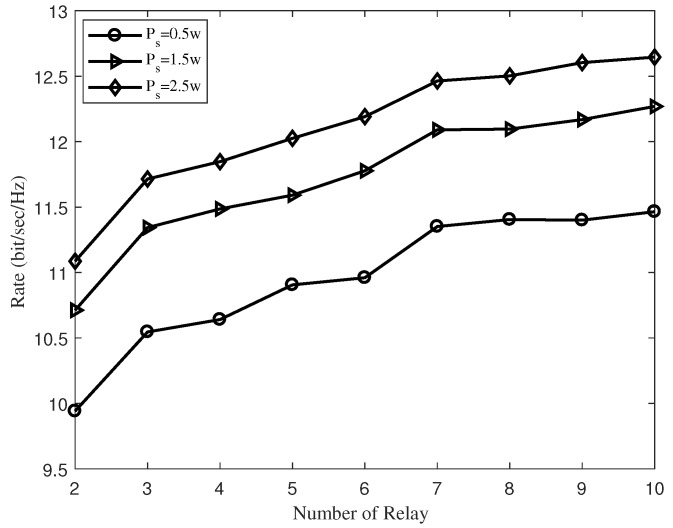
The transmission rate versus the number of relays with different source transmit power $P_{s}$.

In Figure 5, we exhibit the transmission rate versus the number of relays with different $P_{s}$. It is obvious that the increase of the source’s transmitted power results in the increase of the transmission rate. Specifically, the improvement in the transmission rate from $P_{s}=0.5$ W to $P_{s}=2.5$ W with four relays for the proposed algorithm is approximately 1.23 bit/s/Hz. Meanwhile, the simulation result in Figure 5 verifies the conclusion that the system with more relays has a larger transmission rate under the same source’s transmitted power. In addition, it is noteworthy that the improvement in the transmission rate diminishes when the number of relays is large enough. This is because the performance of the system is mainly restricted by the source’s transmitted power.

**Figure 6:**
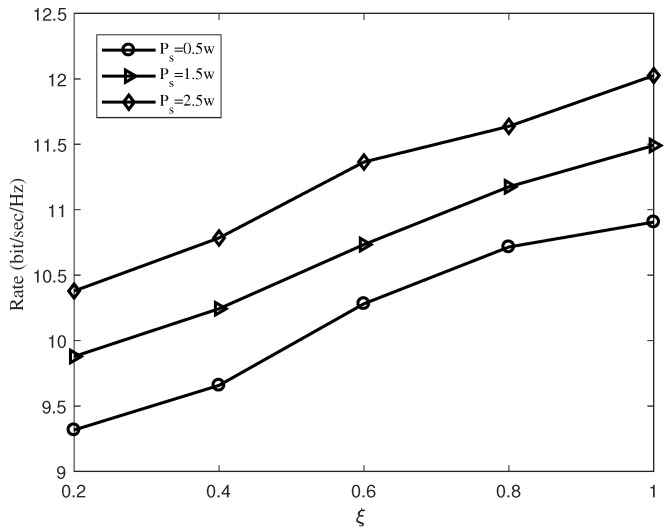
The transmission rate versus the energy conversion efficiency ξ.

In Figure 6, we exhibit the transmission rate versus the energy conversion efficiency ξ with five relays. Considering the different situations, the energy conversion efficiency of the relay will also be different. Therefore, it is necessary to explore the effects of different energy conversion efficiencies on our algorithm, which can prove the reliability of the algorithm. It is clear that as ξ is gradually adjusted from 0.2 to 1, the information transmission rate is increasing. In particular, when ξ is varied from 0.2 to 1, the transmission rate is improved about 17.2%, 16.4%, 15.8% for $P_{S}=0.5$ W, $P_{S}=1.5$ W and $P_{S}=2.5$ W, respectively.

**Figure 7:**
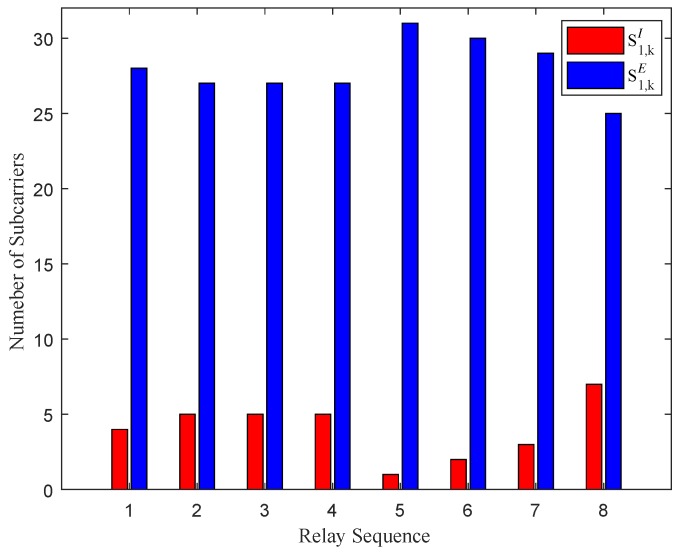
Subcarrier allocation for energy harvesting and information decoding in the first timeslot.

**Figure 8:**
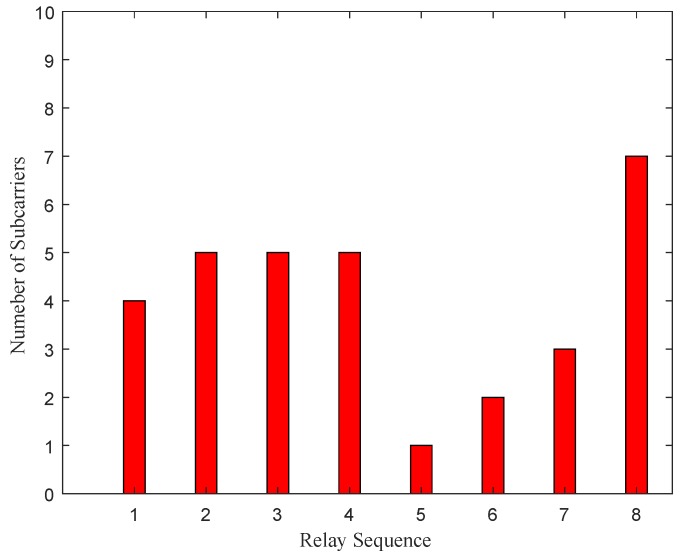
Subcarrier allocation for signal forwarding in the second timeslot.

Figure 7 shows the subcarrier allocation for energy harvesting and information decoding in the first timeslot. It is clear that more subcarriers are utilized for energy harvesting, which goes toward meeting the energy requirements for relays to forward information in the second timeslot. Figure 8 shows the subcarrier allocation for signal forwarding in the second timeslot. We can find that that due to the difference in subcarrier channels, the subcarriers allocated for relays to forward signals are different. For instance, the 5th relay is assigned only one subcarrier to transfer information while the 8th relay is allocated seven subcarriers. Therefore, it can be expected that if a subpar channel is found to exist, the system will no longer allocate the subcarrier to this channel. Moreover, by comparing Figure 7 and Figure 8, it can be observed that the more subcarriers are assigned by the relay for information decoding, the larger proportion of subcarriers are allocated to forward re-encoded information.

## 6. Conclusions

In this paper, we study the transmission rate optimization problem for a dual-hop multi-relay OFDM based IoT network, where DF relay supports SWIPT technique. The joint optimization of power and subcarrier allocation aims to maximize the transmission rate. By adopting the time-sharing strategy, we convert the mixed integral program problem with multi-constraint to the convex optimization problem which can be solved by Lagrange dual method. The effects of the source transmit power, the number of relays and the energy conversion efficiency on the performance of the proposed algorithm have been investigated. Simulation results demonstrate that the proposed algorithm has higher transmission rate compared with other benchmark algorithms when ensuring the relay has no additional energy supply. In particular, when $P_{s}=0.5$ w, our proposed algorithm achieves the improvement in the transmission rate about 2.8%, 3.4% and 43% over the Algorithms 2–4.

In the future work, we will consider the relay selection optimization, in which not all of the relays will be allocated subcarriers to forward information. Moreover, We will study the amplify-and-forward relaying protocol in SWIPT enabled multi-relay IoT system by considering the source and destination direct link. Besides, it is also an interesting direction to study a two-way multi-relay SWIPT enabled IoT system which is able to achieve higher spectrum utilization.
